# The Co-evolution of Neuroimaging and Psychiatric Neurosurgery

**DOI:** 10.3389/fnana.2016.00068

**Published:** 2016-06-22

**Authors:** Timothy G. Dyster, Charles B. Mikell, Sameer A. Sheth

**Affiliations:** Functional and Cognitive Neurophysiology Laboratory, Department of Neurological Surgery, Columbia University Medical Center, New York Presbyterian HospitalNew York, NY, USA

**Keywords:** neuroimaging, magnetic resonance imaging (MRI), psychiatric neurosurgery, cingulotomy, capsulotomy, obsessive-compulsive disorder (OCD), major depressive disorder (MDD)

## Abstract

The role of neuroimaging in psychiatric neurosurgery has evolved significantly throughout the field’s history. Psychiatric neurosurgery initially developed without the benefit of information provided by modern imaging modalities, and thus lesion targets were selected based on contemporary theories of frontal lobe dysfunction in psychiatric disease. However, by the end of the 20th century, the availability of structural and functional magnetic resonance imaging (fMRI) allowed for the development of mechanistic theories attempting to explain the anatamofunctional basis of these disorders, as well as the efficacy of stereotactic neuromodulatory treatments. Neuroimaging now plays a central and ever-expanding role in the neurosurgical management of psychiatric disorders, by influencing the determination of surgical candidates, allowing individualized surgical targeting and planning, and identifying network-level changes in the brain following surgery. In this review, we aim to describe the coevolution of psychiatric neurosurgery and neuroimaging, including ways in which neuroimaging has proved useful in elucidating the therapeutic mechanisms of neuromodulatory procedures. We focus on ablative over stimulation-based procedures given their historical precedence and the greater opportunity they afford for post-operative re-imaging, but also discuss important contributions from the deep brain stimulation (DBS) literature. We conclude with a discussion of how neuroimaging will transition the field of psychiatric neurosurgery into the era of precision medicine.

## Psychiatric Surgery in the Pre-Imaging Era

Long before the advent of neuroimaging, psychiatric neurosurgery was born. At the time, knowledge about the human brain’s functional anatomy was limited, and surgeons relied heavily on contemporary theory and scientific instinct to select neuroablative targets. In 1888, when Dr. Gottlieb Burckhardt removed the left frontotemporal cerebral cortex in six patients with psychiatric diagnoses, his surgical plan was based on results from animal studies and evidence of hypertrophic gyri in psychiatric patients (Burckhardt, 1891; Joanette et al., 1993). Burckhardt hypothesized that different regions of the cerebral cortex were responsible for different behavioral functions, and termed these localizations “psychic domains” (Burckhardt, 1891; Stone, 2001). He speculated aberrant activity in specific domains was responsible for psychiatric pathology, and that ablation of those regions would cure psychiatric disease. Burckhardt’s operations, which lasted only a few hours and were performed with a sharp spoon (Burckhardt, 1891; Stone, 2001), were considered ethically dubious by his contemporaries (de Meuron, 1949; Joanette et al., 1993). Nonetheless, Burckhardt’s work represented an effort to translate contemporary neuroscience into neurosurgical technique, and thus set the stage for further co-mingling of these nascent fields.

Half a century later, in the 1930s, Moniz developed the “prefrontal leucotomy,” a procedure that involved coring small regions of the frontal lobes (Moniz, 1937). His surgical plan was inspired by emerging evidence of calmed behavior in animals after resection of the anterior frontal cortex, although performing a procedure based on this evidence was considered controversial (Munoz and Iniguez, 1949). The prefrontal leucotomy had highly inconsistent results, but Moniz (1937) reported improvement in some patients. In 1949, he was awarded the Nobel Prize for the leucotomy, although his body of work was also notable for pioneering cerebral angiography (Moniz, 1933). Freeman (1957), an American neurologist, learned from Moniz and promoted the modified “lobotomy” procedure in the United States. His infamous “icepick” lobotomies, formally the transorbital lobotomy (Freeman, 1949), fell out of favor and maligned the developing field due to overuse, unacceptable long-term morbidity, and Freeman’s brazen self-promotion (Diefenbach et al., 1999).

In the following decades, psychiatric neurosurgery became safer and more precise with the introduction of stereotactic techniques. Developed in the early 1900s by Horsley and Clarke (1908) the stereotactic frame was first used to target specific locations in animal brains described by a Cartesian coordinate system. Spiegel et al. (1947) translated the technology to humans, and performed the first stereotactic human neurosurgical procedure. Using the “Horsley apparatus”, Spiegel and Wycis were able to minimize injury to the cerebral cortex and white matter while performing a stereotactic medial thalamotomy to treat a behavioral disorder.

In addition to improved safety and precision, stereotactic neurosurgery offered increased reproducibility, and therefore an opportunity to evaluate a surgical target’s efficacy when used across many patients. In 1962, Ballantine performed the first stereotactic bilateral cingulotomy (Ballantine et al., 1967, 1987), based on Fulton’s (1951) proposal that the cingulate cortex should be considered as a surgical target. In the following 4 years, Ballantine et al. (1967) performed an additional 94 such procedures. The cingulotomy target used by Ballantine et al. (1967) was based on contemporary theory and on the work of Foltz and White (1962), who performed the procedure for intractable pain but had observed their best outcomes in patients with co-morbid anxiety or depression (Foltz and White, 1962; Ballantine et al., 1967). Similarly, in 1949, Talairach based his anterior capsulotomy target, the anterior limb of the internal capsule (ALIC), on the hypothesis that the internal capsule had connections with the “limbic system,” a circuit proposed by Papez (1937) that included the cingulum bundle, hippocampus, thalamus, and hypothalamus and was believed to mediate emotional experience and expression (Papez, 1937; Talairach et al., 1949). Thus, by the mid-twentieth century, the field had made significant advances since its advent, but strong evidence to explain the mechanisms underlying neuroablative targets remained elusive.

## Psychiatric Neurosurgery and the Advent of Neuroimaging

The introduction of neuroimaging dramatically expanded the repertoire of techniques for explaining how neuroablation treated psychiatric disease. Neuroimaging made it possible to demonstrate where functional domains localized within the brain and to analyze structural differences between patients and healthy controls. Using these newly available methods, investigators could show that surgical targets selected decades earlier based on theory and trial-and-error were actually at sites that localized functions impaired in psychiatric disease or that exhibited structural correlates of a patient’s functional pathology. To appreciate the neuroimaging data that shows why early ablative procedures such as the capsulotomy and cingulotomy are efficacious, one must first understand the functional and neurobiological models of the psychiatric disorders that the procedures are being used to treat.

### Functional Models of Psychiatric Disorders

Within the last few decades, interest has grown in conceiving psychiatric disorders in a spectrum-based model, with emphasis on impairment in functional domains (Castle and Phillips, 2006). This model offers the advantage that a single functional domain may span multiple classic psychiatric diagnoses, and descriptions using several functional domains can help to subtype patients within a single diagnostic class (McElroy et al., 1994; Castle and Phillips, 2006). For example, the classic diagnostic criteria for obsessive-compulsive disorder (OCD) requires the presence of clinically significant obsessions (intrusive and unwanted thoughts), compulsions (ritualistic behavior or thoughts), or both (American Psychiatric Association, 2013). In a functional domain-based model, however, OCD might be conceived as dysfunction in such domains as cognitive control, attention, reward sensitivity, learning, memory, fear response, and mood stability (Hollander et al., 2012). Evidence for a possible deficit in memory confidence, or the ability to determine whether a recalled memory is true, also characterizes the disorder (MacDonald et al., 1997).

Similarly, the classic criteria for major depressive disorder (MDD) include anhedonia or depressed mood, with the addition of at least four more symptoms such as: change in sleep pattern, psychomotor status, or concentration; fatigue; feelings of worthlessness; or recurrent thoughts of death (American Psychiatric Association, 2013). In a functional domain-based model, MDD might be conceived as a dysfunction of cognitive control, learning, mood stability, and assigning value to self, actions, and objects (Austin et al., 1992; Veiel, 1997; American Psychiatric Association, 2013). OCD and MDD serve as prominent examples because they represent the majority of indications for psychiatric neurosurgical procedures, but many other examples exist, including schizophrenia (Mikell et al., 2016), autism (Sturm et al., 2012; Rutishauser et al., 2015; Sinha et al., 2015), post-traumatic stress disorder (Langevin et al., 2010, 2016), eating disorders (Halpern et al., 2008; Lipsman et al., 2013), and others.

Thus understanding the mechanism of stereotactic neuromodulatory procedures requires understanding the networks involved with these functional domains. This relationship between function and treatment is of course not limited to surgical procedures. Indeed, the National Institute of Mental Health (NIMH) has recently proposed a strategy for classifying all mental health disorders based on neurobiological measures as described above, termed the Research Domain Criteria (RDoC) project (Insel et al., 2010). The NIMH’s strategic plan notes that the classification scheme for psychiatric disorders, the Diagnostic and Statistical Manual (DSM), is based on clinical observation of certain symptom clusters. While this scheme has been in use for decades, it does have important shortcomings, most notably that its diagnoses are not necessarily coherent pathophysiological entities, but rather heterogeneous collections of symptoms that at times describe a syndrome, rather than define a disease process (Clark et al., 1995; Cross-Disorder Group of the Psychiatric Genomics Consortium, 2013; Cuthbert, 2014). It is therefore often difficult to ascribe for these diagnostic entities a neurobiological substrate that can be systematically subjected to research, and consequently difficult for the research to be translated back into substrate-directed treatments.

The RDoC system, on the other hand, attempts to project observable symptoms and measurable variables onto a set of axes (“Domains”, with sub-categories of “Constructs” and “Subconstructs”) defined in terms of functional neurobiological systems. A phenomenological diagnosis such as “OCD” would thus be re-classified according to dysfunction of fundamental constituent aspects of behavior and circuitry. Some of the key aspects of dysfunction in OCD include performance monitoring, response inhibition, and goal selection, all of which are classified under the RDoC construct of Cognitive Control (which is contained within the larger domain of Cognitive Systems). OCD may also project onto domains of Negative Valence Systems (Sustained Threat construct) and Positive Valence Systems (Reward Learning construct). Similarly, the diagnosis “MDD” may involve dysfunction in domains of Positive Valence Systems (Approach Motivation), Negative Valence Systems (Loss), Cognitive Systems (Cognitive Control), Arousal, and perhaps others.

Another specifically mentioned motivation for creating the RDoC classification was to align the taxonomy of mental illness with neuroscience research (Insel et al., 2010; Cuthbert, 2014). Because the domains and constructs within them comprise defined fields of research, they may be studied using various “Units of Analysis,” including behavioral, genetic, physiological, and especially relevant to this discussion, imaging studies. Thus the recognition that advanced neuroimaging has contributed to improvements in psychiatric neurosurgery runs parallel to, and in some ways represents a microcosm of, broader trends to align the diagnosis and treatment of mental health disorders with measurable biomarkers.

### Contributions by the Pre-Frontal Cortex and Other Brain Regions to the Functional Domains Impaired in OCD and MDD

Through the use of neuroimaging, the functional domains impaired in psychiatric disease, including cognitive control, attention, reward sensitivity, memory, learning, fear response, and assignment of value, have been shown to involve specific regions of the brain relevant to psychiatric neurosurgery, including the cingulate cortex.

The cingulate cortex, especially the dorsal anterior cingulate cortex (dACC), has been linked to several functional domains relevant to OCD and MDD, including fear response, anxiety, reward response, and memory. In a neuroimaging meta-analysis, the dACC demonstrated increased activity in patients who underwent fear or anticipatory anxiety conditioning (Mechias et al., 2010), suggesting a role for the cingulate in fear response, anxiety, and threat appraisal, three of the functional domains impaired in OCD. Similarly, a meta-analysis of 171 functional magnetic resonance imaging (fMRI) studies revealed activation peaks in the dACC during tasks involving reward responses, memory, and negative outcomes, further underscoring the dACC’s role in functional domains impaired in OCD and MDD (Beckmann et al., 2009). In a domain-based model of psychiatric disease, these results identify dACC as a key node in psychiatric pathology, and accordingly, as a possible therapeutic target.

Cognitive control, the cognitive process used to overcome interference or habitual responses, is another cognitive domain impaired in OCD and MDD, and has been linked to the cingulate cortex. Patients who are completing interference tasks demonstrate increased activity in the anterior cingulate cortex (ACC), dorsolateral prefrontal cortex (DLPFC), inferior frontal gyrus (IFG), and posterior parietal cortex (PPC; Nee et al., 2007). In addition to its role in cognitive control, anatomical, physiological, and functional data link the midcingulate cortex to negative affect and pain (Shackman et al., 2011). Anatomically, the cingulate cortex represents an intersection of fibers from the amygdala, thalamus, striatum, and other regions, and it has been proposed that its core function is to use input from these connected regions to determine an optimal course of action when the best course of action is uncertain (Shackman et al., 2011). Similarly, cognitive neuroscientists have proposed an overarching function for the dACC, termed “expected value of control” (Shenhav et al., 2013). In this model, the dACC determines whether to exert cognitive control by integrating three pieces of information: the predicted benefit of exerting cognitive control, the amount of control required to achieve the predicted benefit, and the expected cost in cognitive effort. Expected value of control provides another important link between the cingulate cortex and psychiatric pathology, as impaired ability to perform the cost-benefit calculation for cognitive control could cause some of the symptoms seen in OCD and MDD. Unsurprisingly, cognitive control is considered a relevant RDoC construct for both pathologies.

Thus the cingulate cortex plays a clear role in domains impaired in psychiatric disease. However, evidence suggests other brain regions are also linked to relevant functional domains. The PFC has been implicated in cognitive control (Miller and Cohen, 2001), reward sensitivity (Ridderinkhof et al., 2004), memory and attention (Kane and Engle, 2002), fear (Mechias et al., 2010; Marek et al., 2013), memory confidence, (Chiang et al., 2014) and assignment of value (Murray et al., 2011). The thalamus has been implicated in memory and attention (de Bourbon-Teles et al., 2014), and has been shown to interact with the amygdala to produce the fear response (LeDoux et al., 1990; Ciocchi et al., 2010). The amygdala plays roles in fear (Davis, 1992), learning (Morris et al., 1998), and memory (Hamann et al., 1999). The hippocampus is crucial for memory formation (Tulving and Markowitsch, 1998), and the role of the nucleus accumbens in reward sensitivity is well-established (Ikemoto and Panksepp, 1999). In summary, while the list is ever expanding, the functions impaired in OCD and MDD localize to several specific areas of the brain, including the dACC, PFC, thalamus, amygdala, hippocampus, and nucleus accumbens.

### How Structural and Functional Imaging Inform Neurobiological Models of OCD and MDD

#### Obsessive-Compulsive Disorder

**Figure 1:**
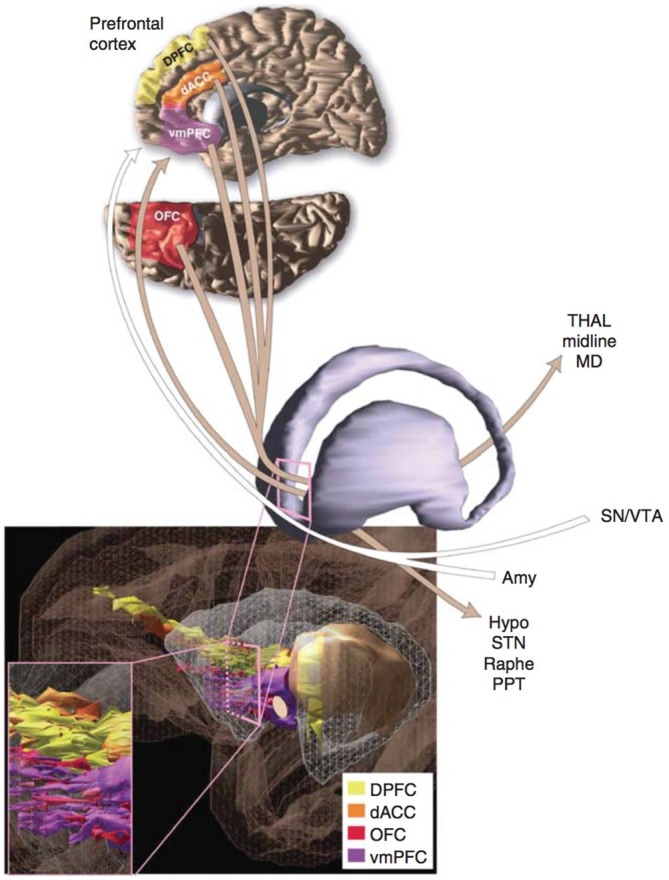
**A schematic representation of the network passing through the ventral portion of the anterior limb of the internal capsule (ALIC) that connects prefrontal cortex to several additional regions of the brain.** Many of the depicted regions contribute to the proposed cortico-striato-thalamo-cortical (CSTC) loop that may underlie obsessive-compulsive disorder (OCD). Additional abbreviations used in the figure: DPFC, dorsolateral prefrontal cortex; dACC, dorsal anterior cingulate cortex; vmPFC, ventromedial prefrontal cortex; THAL, midline and mediodorsal (MD) nuclei of the thalamus; SN/VTA, substantia nigra and ventral tegmental area; Amy, amygdala; Hypo, hypothalamus; STN, subthalamic nucleus; Raphe, raphe nuclei; OFC, orbitofrontal cortex; PPT, pedunculopontine tegmental nucleus. Reprinted with permission from Greenberg et al. (2010b); Macmillan Publishers Ltd.

The current model of OCD’s neurobiology was informed by a general neuroanatomical concept introduced in the late 20th century. In the 1980s, Alexander proposed that the cerebral cortex and subcortical structures might be organized into cortico-striato-thalamo-cortical (CSTC) loops (Alexander et al., 1986). Modell et al. (1989) extended this concept to psychiatric pathology, proposing that a CSTC loop involving the orbitofrontal cortex (OFC), basal ganglia, limbic striatum, and thalamus underlay OCD (Figure 1). In the model, the symptoms of OCD resulted from overactivity of the frontothalamic portion of the loop and failure of the limbic striatum to inhibit this activity (Modell et al., 1989). The caudate and cingulate were hypothesized to also play a central role in OCD pathogenesis (Modell et al., 1989; Insel, 1992). Thus the model made several critical predictions about patients with OCD: first, there should be overactivity in the frontal and thalamic structures; second, evidence for dysfunction in the limbic striatum should be present; third, structural changes, if present, should predominately exist in the OFC/PFC, cingulate, and caudate.

In the years after this model of OCD was proposed, new methods for imaging analysis that could test the critical predictions of the model were developed. Magnetic resonance diffusion tensor imaging (DTI), a technique that exploits the diffusion properties of water to determine fiber tract orientation (Basser et al., 1994), was combined with computational tractography methods to estimate neural fiber pathways in the white matter. Later modifications allowed the technique to evaluate gray matter structures, both for segmentation within the gray matter and to analyze the connectivity between different gray matter structures (Behrens et al., 2003).

These and other emerging imaging techniques provided *in vivo* evidence of structural abnormalities in patients with OCD. Neuroimaging studies indicated that patients with OCD had reduced PFC volume (Szeszko et al., 1999; Alonso et al., 2001; Choi et al., 2004; Kang et al., 2004; Pujol et al., 2004; Atmaca et al., 2006), reduced ACC volume (Szeszko et al., 2004), and changes in the size of the caudate nucleus (Scarone et al., 1992; Robinson et al., 1995). DTI studies suggested abnormalities in the white matter microstructure (Szeszko et al., 2005), left-lateralized asymmetry in cingulate connectivity, and left-lateralized asymmetry in connectivity between the thalamus and PFC (Chiu et al., 2011). Neuroimaging evidence also suggested structural changes in the amygdala (Szeszko et al., 1999; Kwon et al., 2003) and hippocampus (Kwon et al., 2003). These OCD-associated structural changes in the PFC, ACC, caudate, thalamus, amygdala, and hippocampus were consistent with the loop model of OCD and thus provided support for this proposed model.

In addition to evidence of structural change, emerging imaging techniques provided evidence of functional abnormalities in patients with OCD. Shortly after the development of fMRI, researchers evaluated neural activity in patients with OCD in response to stimuli that were known triggers for the patients’ symptoms (Breiter et al., 1996). Compared to healthy controls, patients with OCD showed increased activation in lateral PFC, medial OFC, ACC, temporal and insular cortex, caudate, amygdala, and lenticulate nuclei when presented with the trigger stimulus. This pattern of functional change, which involved several frontal and limbic structures, corroborated existing structural evidence and further supported the loop model of OCD.

#### Major Depressive Disorder

The neurobiological mechanisms of normal mood and mood disturbances remain areas of active scientific investigation (Nestler et al., 2002). The proposed mechanisms for depression exist on multiple levels of analysis, and many are not mutually exclusive. Among the possibilities are dysfunction in the stress axis, altered glutamergic neurotransmission, reduced GABAergic transmission, abnormal circadian rhythms, and thyroxine abnormalities (Belmaker and Agam, 2008). In this review, we focus on how dysfunction in specific brain structures and circuits might underlie MDD, as this model of MDD has been strongly informed by neuroimaging.

Early neurobiological models of MDD were influenced by the CSTC loop model, similar to early models of OCD. Initially, data from PET scans implicated the cortico-basal ganglia circuits, OFC, mesencephalon, and basotemporal limbic regions in MDD (Mayberg, 1994). These data, combined with clinical observation, informed a three “compartment” model of MDD (Mayberg, 1997). In the three-compartment model, a dorsal compartment, comprising cortical and midline limbic structures (dACC, DLPFC, inferior parietal cortex, and striatum), and a ventral compartment, comprising paralimbic regions (hypothalamus, insula, subgenual cingulate, and brainstem), were regulated and kept in balance by a third region, the rostral cingulate. Disturbances in mood represented failure of the cingulate to coordinate the other compartments. This unbalanced activity caused the dorsal compartment to produce attentive-cognitive symptoms, and the ventral compartment to produce vegetative-somatic symptoms.

**Figure 2:**
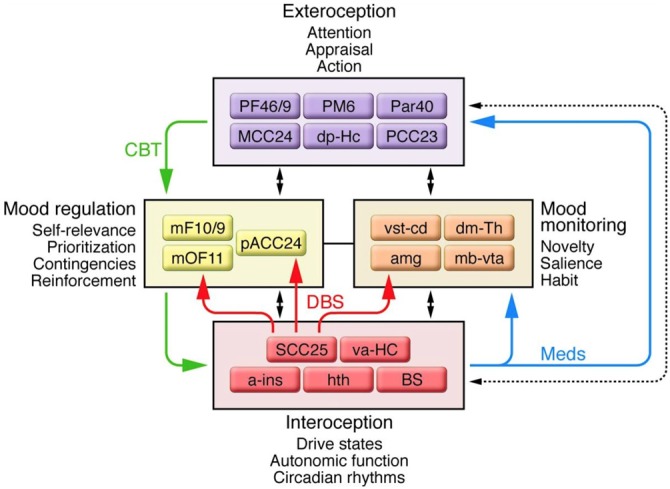
**A schematic representation of the network of connections between cortical, limbic, and paralimbic regions proposed to underlie major depressive disorder (MDD), and the four main functional compartments of the model, each of which represents a functional domain impaired in MDD and comprises a subset of brain regions that demonstrate robust anatomical connections to one another.** Black arrows identify anatomical connections between compartments. Solid colored arrows identify connections that are proposed to mediate efficacy of a specific treatment: green indicates cognitive behavioral therapy; blue indicates pharmacotherapy; red indicates deep brain stimulation (DBS) at subcallosal cingulate. Additional abbreviations used in the figure: a-ins, anterior insula; amg, amygdala; dm-Th, dorsomedial thalamus; dp-Hc, dorsal-posterior hippocampus; mb-vta, midbrain-ventral tegmental area; mF10/9, medial frontal cortex BA10 and BA9; mOF11, medial orbital frontal cortex BA11; Par40, parietal cortex BA40; PF46/9, PFC BA46 and BA9; PM6, premotor cortex BA6; va-HC, ventral-anterior hippocampus; vst-cd, ventral striatum-caudate. Republished with permission of American Society for Clinical Investigation, from Mayberg (2009); permission conveyed through Copyright Clearance Center, Inc.

Advances in neuroimaging provided evidence that complicated the three-compartment model of MDD. Structural and functional MRI demonstrated changes in the cingulate cortex, PFC, amygdala, hippocampus, hypothalamus, and nucleus accumbens associated with MDD (Sheline et al., 1999; Nestler et al., 2002; Gutman et al., 2009; Mayberg, 2009; Murray et al., 2011; Patel et al., 2013). In 2009, an updated model involving four regions was proposed (Figure 2; Mayberg, 2009). The four-region model included a more comprehensive set of brain regions; however, the “dorsal compartment” and “ventral compartment” from the prior model approximated the new model’s “exteroceptive region” and “interoceptive region,” respectively. The exteroceptive region controlled cognitive function (e.g., attention, appraisal, action), while the interoceptive region controlled visceral-motor function (e.g., drive states, autonomic function, circadian rhythms). The four-region model maintained that mood disturbances resulted from a lack of coordination between exteroceptive and interoceptive activity. Sustained loss of coordination produced major depressive episodes. Two regions in the model were new: a mood-monitoring region (comprising subcortical regions such as the dorsomedial thalamus, amygdala, and basal ganglia), and a mood regulation region (comprising regions of the medial frontal cortex including the rostral ACC). The mood-monitoring region processed both emotional and non-emotional stimuli, while the mood regulation region mediated cognitive control and overt control of emotional state. Thus in its final form, the model included the striatum, amygdala, dorsomedial thalamus, and cingulate cortex, among other regions (Mayberg, 2009), constituting a complex cortico-limbic-thalamic-striatal network.

### The Cingulotomy and Capsulotomy: Target Anatomy and Connectivity

#### Cingulotomy

Currently, cingulotomy is mostly performed for severe, refractory OCD (Bourne et al., 2013; Sheth et al., 2013) and MDD (Shields et al., 2008; Steele et al., 2008), although chronic neuropathic pain (Viswanathan et al., 2013; Patel et al., 2015) and other indications also exist (Kanaka and Balasubramaniam, 1978; Baer et al., 1994). The cingulate cortex is a curved band of cortex that overlies the corpus callosum just off the midline, and the cingulotomy specifically targets a region approximately 15–20 mm posterior to the anterior-most point of the frontal horn of the lateral ventricles (Kim et al., 2003; Yang et al., 2014), placing it in the “anterior midcingulate,” a region associated with cognitive control functions (Shackman et al., 2011; Cavanagh and Shackman, 2015).

The cingulotomy lesion also typically extends into the cingulum bundle, the white matter pathway immediately ventral to the cingulate cortex, and disrupts cingulate fibers projecting to other regions of the brain (Jellison et al., 2004; Steele et al., 2008). Therefore, to fully understand the cingulotomy’s mechanism requires an understanding of the cingulate’s connectivity. Anatomical tract-tracing in nonhuman primate demonstrates cingulate fibers running in distinct bundles to the striatum, corpus callosum, internal capsule, external capsule, OFC and DLPFC (Heilbronner and Haber, 2014). In healthy human subjects, DTI demonstrates cingulate fibers to PFC, amygdala, striatum, hypothalamus, and parietal cortex (Beckmann et al., 2009). The cingulate’s rich network notably includes several regions with known structural and functional associations to MDD and OCD. Considering these connectivity data and the cingulate’s proposed role in cognitive control, a plausible mechanism for the cingulotomy’s efficacy emerges: the cingulotomy ablates neural regions and tracts that underlie OCD and MDD.

#### Capsulotomy

Capsulotomy (or anterior capsulotomy) has primarily been used to treat severe, refractory OCD (Rück et al., 2008; Kondziolka et al., 2011; Sheehan et al., 2013; Lopes et al., 2014) and MDD (Christmas et al., 2011; Hurwitz et al., 2012), although other indications also exist (Rück et al., 2003; Barbier et al., 2011). The general target for the procedure is in the ventral portion of the ALIC. Initially, capsulotomy lesions were made exclusively using radiofrequency thermal techniques (Talairach et al., 1949), but more recently stereotactic radiosurgery techniques have also been used (Rück et al., 2008; Kondziolka et al., 2011; Sheehan et al., 2013; Lopes et al., 2014). Early reports described a large target region between the anterior and middle third of the ALIC at the level of the foramen of Monro, 10 mm anterior to the anterior commissure, 8 mm superior to the intercommisural plane, and 17 mm from the midline (Kihlström et al., 1997), although similar targets have been reported using slightly different landmarks (Rück et al., 2008; D’Astous et al., 2013). The lesions made at the target were 15–20 mm long and 4–5 mm wide (Kihlström et al., 1997; Aouizerate et al., 2006; Rück et al., 2008; D’Astous et al., 2013). More recent reports describe a smaller target region, closer to the ventral portion of the ALIC, 7–10 mm anterior to the anterior commissure, near the level of the intercommisural plane (Kondziolka et al., 2011; Sheehan et al., 2013; Lopes et al., 2014). These lesions are spherical to slightly oblong, extend less dorsally, and are smaller overall, with a widest diameter of a few to several millimeters.

The internal capsule serves as a conduit for a large bundle of fibers that run to and from the cerebral cortex (Jellison et al., 2004). As with the cingulotomy, to fully understand capsulotomy’s mechanism requires an understanding of the internal capsule’s connectivity. The internal capsule can be subdivided into anterior and posterior limbs, and each limb has a distinct pattern of connectivity. The anterior limb, which is targeted by capsulotomy, lies between the head of the caudate and the lenticular nucleus (Jellison et al., 2004). Tractography reveals projections from the ALIC to cortex, pons and thalamus in the anterior-posterior plane, and projections from the posterior limb into corticospinal, corticobulbar, and corticopontine tracts, mostly in the superior-inferior plane (Jellison et al., 2004). Neuroimaging studies further suggest internal capsule fibers target the PFC, amygdala, hippocampus, nucleus accumbens, thalamus, and cingulate (Behrens et al., 2003; Haber et al., 2006; Gutman et al., 2009). Considering these data, a few important patterns emerge. First, the areas targeted by fibers from the ALIC and the cingulate cortex show significant overlap (Gutman et al., 2009). Further, evidence suggests the reciprocal connections running through ALIC to thalamus and OFC interface with caudate and brainstem, consistent with the CSTC loop model and suggesting the ALIC may be an important loop node (Modell et al., 1989). Finally, fibers projecting through the ALIC come from regions with known structural and functional associations to OCD and MDD, implying a conceivable mechanism, similar to the mechanism of the cingulotomy, for the capsulotomy: the procedure disrupts tracts between neural regions that underlie OCD and MDD.

In summary, a neurobiological mechanism for both cingulotomy and capsulotomy is supported by modern fMRI and structural MRI data. These neuroablative procedures target the regions and tracts that have been shown to functionally and structurally underlie OCD and MDD.

#### Relevant Examples from Deep Brain Stimulation

This review focuses on cingulotomy and capsulotomy over DBS due to neuroablative procedures’ historical role in understanding functional anatomy, longer follow-up times, and ease of re-imaging; however, it is important to briefly discuss DBS as well, as DBS plays an increasingly important role in the treatment of psychiatric disease, including MDD and OCD. DBS targets for MDD have included ventral capsule/ventral striatum (VC/VS; Malone et al., 2009), nucleus accumbens (Bewernick et al., 2012), and subcallosal cingulate (Lozano et al., 2008, 2012; Holtzheimer et al., 2012), whereas targets for OCD have included VC/VS (Greenberg et al., 2006, 2010a; Goodman et al., 2010; Mian et al., 2010), ALIC (Abelson et al., 2005), nucleus accumbens (Denys et al., 2010; Huff et al., 2010), inferior thalamic peduncle (Jiménez-Ponce et al., 2009), and subthalamic nucleus (STN; Mallet et al., 2008; Chabardès et al., 2013).

The VC/VS is among the most well-studied DBS targets for the treatment of OCD (Greenberg et al., 2006, 2010a; Goodman et al., 2010; Mian et al., 2010; Dougherty et al., 2016). In 1999, four patients with severe OCD underwent bilateral electrode implantation rostral to the anterior commissure with extension into the VC/VS. The results with stimulation were modest but promising (Nuttin et al., 1999), and the target’s efficacy continued to be evaluated in small controlled- and open-studies. In one open-label series, eight patients with severe or resistant OCD who underwent implantation were followed to three-years of follow up, and demonstrated an average improvement from severe to moderate, with four of the eight showing ≥35% improvement in Yale-Brown Obsessive Compulsive Scores (YBOCS) severity, and two additional patients showing 25–35% improvement in YBOCS severity (Greenberg et al., 2006). Over time, the VC/VS target was refined and moved to a more posterior location, where the anterior capsule, anterior commissure, and posterior ventral striatum intersected and CSTC circuit fibers were thought to be more densely packed (Greenberg et al., 2010a). With this posterior shift, response rates to the procedure improved (Greenberg et al., 2010a). In 2010, a randomized, blinded, staggered-onset study reported ≥35% improvement in YBOCS in four out of six patients, with no significant responses during the sham period. The evolution of the VC/VS target further underscores the important interplay between understanding the neurobiological substrate of psychiatric disease and selecting procedural targets. When a psychiatric process is better understood at the neurobiological level, hypothesis-driven target selection and refinement become possible, which, in turn, have the potential to meaningfully improve clinical outcomes and patient care.

## The Future of Psychiatric Neurosurgery and Neuroimaging

Neuroimaging has helped describe a growing list of functions localized to the cingulate cortex, the internal capsule, and their networks. Perhaps unsurprisingly, this list includes many of the functional domains impaired in the psychiatric diseases treated by cingulotomy and capsulotomy. Moreover, neuroimaging has revealed new connections and confirmed known connections between the cingulate, internal capsule, and other regions of the brain. In many cases, the nodes of these networks are the same regions that demonstrate structural and functional changes in the setting of psychiatric pathology, providing additional rationale for the efficacy of cingulotomy and capsulotomy.

In the present day, nearly all fields of medicine are trending toward the use of biomarkers to practice more personalized, patient-specific, “precision medicine” (Mirnezami et al., 2012), and the approach to managing psychiatric illness is no exception to this trend (Costa e Silva, 2013). This transition offers neuroimaging a new role in the realm of psychiatric neurosurgery. Whereas in the past neuroimaging was used to retrospectively clarify procedural mechanisms, in the future, the same modalities will prospectively inform patient-specific clinical management.

Neuroimaging makes it possible to evaluate patient-specific regional volume and connectivity. Thus one way in which neuroimaging may inform patient-specific clinical management is via the discovery of biomarkers for predicting outcomes in psychiatric neurosurgery. For example, a recent retrospective study analyzed preoperative T1 and diffusion MRI sequences for 15 patients who underwent cingulotomy, of whom only eight had responded to the procedure (Banks et al., 2015). Using voxel based morphometry and probabilistic tractography to analyze the area immediately anterior to the lesion target, the investigators found patients with increased right-sided gray matter signal in ACC exhibited poorer clinical response, while patients with decreased gray matter signal in the same region exhibited improved clinical response. Improved clinical response was also correlated with increased right-sided connectivity between the lesion site and the caudate, putamen, thalamus, pallidum, and hippocampus. These results suggest interplay between individual neuroanatomical variation and clinical response to cingulotomy, and therefore that patient-specific neuroanatomical markers might be useful for predicting clinical outcome. While this analysis was restricted to cingulotomy, it is conceivable that similar findings may eventually be described in other neuroablative procedures.

**Figure 3:**
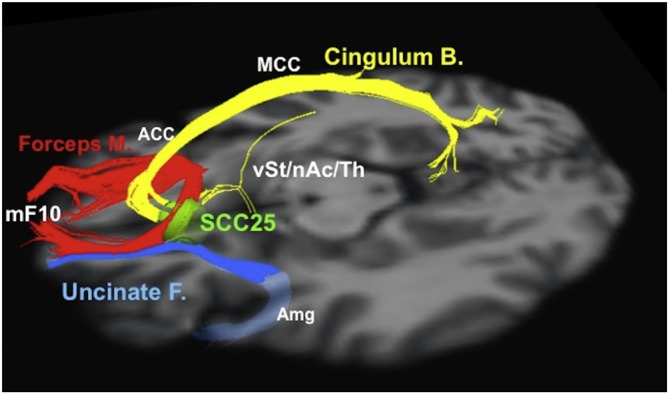
**Tractographic representation of a subcallosal cingulate fiber bundle optimal for DBS.** Probabilistic tractography and similar techniques make it possible to evaluate patient-specific network connectivity when creating surgical plans. Using this information, physicians are able to select individualized stimulation targets at points where critical white matter tracts intersect. Red: forceps minor. Blue: uncinate fasciculus. Yellow: cingulate bundle. ACC, anterior cingulate cortex; Amg, amygdala; Cingulum B., cingulum bundle; Forceps M., forceps minor; MCC, middle cingulate cortex; mF10, medial frontal (Brodmann area 10); nAc, nucleus accumbens; SCC25, subcallosal cingulate cortex (Brodmann area 25); Th, thalamus; Uncinate F., uncinate fasciculus; vSt, ventral striatum. Reprinted from Riva-Posse et al. (2014); Copyright with permission from Elsevier; permission conveyed through Copyright Clearance Center, Inc.

Neuroimaging analysis techniques are also emerging as tools to refine surgical plans and select surgical targets. For example, neuroimaging and other data were used to select the “subgenual” or “subcallosal” cingulate (SCC), located near Brodmann area 25, as a DBS target for depression (Mayberg et al., 1999, 2005; Seminowicz et al., 2004; Mayberg, 2009). The early results from stimulation at SCC were promising, but highly variable, with reported 12-month response rates ranging from 29% to 62% in different series (Lozano et al., 2008, 2012; Hamani et al., 2009; Kennedy et al., 2011). Neuroimaging analysis revealed that the contact locations relative to neuroanatomical landmarks did not explain the differences in patient response (Hamani et al., 2009); however, tractographic analysis for a series of 16 patients who underwent DBS stimulation at SCC revealed that all responders shared bilateral pathways from their activation volumes to the medial frontal cortex, rostral and dorsal cingulate cortex, and subcortical nuclei; conversely, non-responders did not consistently show these connections (Riva-Posse et al., 2014). These results suggest tractography may be useful for optimizing electrode implantation on a patient-by-patient basis using patient-specific anatomical connectivity (Figure 3; Riva-Posse et al., 2014). Prospective trials are currently underway to further test these imaging-driven hypotheses for surgical targeting (^1^NCT00367003, NCT01984710). Similarly, tractography has helped identify the medial forebrain bundle as a node in a subcortical emotional system that may mediate elements of addiction and depression (Coenen et al., 2011). Based on these data, a trial is currently underway to evaluate a novel DBS for depression target, located lateral to the ventral tegmental area in the midbrain, where the superolateral medial forebrain bundle branch emerges from the main medial forebrain bundle (^1^NCT01095263).

In summary, the emerging literature surrounding neuroimaging biomarkers may forecast an era of “precision surgery,” in which patient-specific neuroanatomy, elucidated by neuroimaging analysis, is used to refine surgical targets (Rück et al., 2008), patient selection (Banks et al., 2015), and electrode implantation (Riva-Posse et al., 2014). Undoubtedly, new and innovative uses of neuroimaging in psychiatric neurosurgery will continue to emerge.

## Conclusion

The field of psychiatric neurosurgery has co-evolved with modern neuroimaging modalities. However, the relationship between psychiatric neurosurgery and neuroimaging has shifted dramatically since the field’s advent. Whereas neuroimaging was once used to retrospectively clarify procedural mechanisms, in the future, the same modalities will directly inform clinical decision-making, and, perhaps, will usher in an era of “precision surgery” for psychiatric disease.

## Author Contributions

TGD, CBM, and SAS made substantial contributions to the conception and design of the work, drafted the work and revised it critically, gave final approval of the version to be published, and agree to be accountable for all aspects of the work in ensuring that questions related to the accuracy or integrity of any part of the work are appropriately investigated and resolved.

## Conflict of Interest Statement

The authors declare that the research was conducted in the absence of any commercial or financial relationships that could be construed as a potential conflict of interest.

## Footnotes

^1^www.clinicaltrials.gov
